# Kernel-smoothed permutation for extreme *P-*value estimation in genetic association studies

**DOI:** 10.1093/genetics/iyag119

**Published:** 2026-05-11

**Authors:** Jiayi Bian, Jingjing Wu, M Ethan MacDonald, Lang Wu, Quan Long, Caifeng Li

**Affiliations:** Department of Mathematics and Statistics, University of Calgary, Calgary, Alberta T2N 1N4, Canada; Department of Mathematics and Statistics, University of Calgary, Calgary, Alberta T2N 1N4, Canada; Department of Electrical and Software Engineering, University of Calgary, Calgary, Alberta T2N 1N4, Canada; Hotchkiss Brain Institute, University of Calgary, Calgary, Alberta T2N 1N4, Canada; Alberta Children's Hospital Research Institute, University of Calgary, Calgary, Alberta T2N 1N4, Canada; Department of Biomedical Engineering, University of Calgary, Calgary, Alberta T2N 1N4, Canada; Department of Radiology, University of Calgary, Calgary, Alberta T2N 1N4, Canada; Department of Interdisciplinary Oncology and Department of Genetics, LSU-LCMC Health Cancer Center, School of Medicine, Louisiana State University Health Sciences Center, Louisiana State University, New Orleans, LA 70112, United States; Department of Mathematics and Statistics, University of Calgary, Calgary, Alberta T2N 1N4, Canada; Hotchkiss Brain Institute, University of Calgary, Calgary, Alberta T2N 1N4, Canada; Alberta Children's Hospital Research Institute, University of Calgary, Calgary, Alberta T2N 1N4, Canada; Department of Biochemistry and Molecular Biology, University of Calgary, Calgary, Alberta T2N 1N4, Canada; Department of Medical Genetics, University of Calgary, Calgary, Alberta T2N 1N4, Canada; Department of Mathematics and Statistics, University of Calgary, Calgary, Alberta T2N 1N4, Canada

**Keywords:** *P*-value estimation, test statistic, permutation test, kernel-based density estimation, genetic association studies

## Abstract

In genetic association studies, permutation tests serve as a cornerstone to estimate *P*-values. This is because researchers may design new test statistics without a known closed-form distribution, or the assumption of a well-established test may not hold. However, permutation tests require a vast number of permutations, which is proportional to the magnitude of the actual *P*-values. When it comes to genome-wide association studies where multiple-test corrections are routinely conducted, the actual *P*-values are extremely small, requiring a daunting number of permutations that may be beyond the available computational resources. Existing models that reduce the required number of permutations all assume a specific format of the test statistic to exploit its specific statistical properties. We propose Kernel-smoothed permutation, which is a model-free method universally applicable to any statistic. Our tool forms the null distribution of test statistics using a kurtosis-driven transformation, followed by a kernel-based density estimation. We compared our Kernel-smoothed permutation to Naïve permutation using statistics from known closed-form null distributions. Based on 3 frequently used test statistics in association studies, ie *t*-test, sequence kernel association test, and chi-squared test, we demonstrated that our model reduced the required number of permutations by a magnitude with similar or higher accuracy. Based on a real-world genome-wide association study analysis, we used Crohn's disease cohort to further confirm that our model substantially outperforms the Naïve permutation.

## Introduction

In the hypothesis testing, one needs to compute the *P*-value out of the test statistic using the null distribution (Banerjee et al. 2009). Although this can be done analytically, for many complex situations, especially in genetic association studies, the closed form of the null distribution may not be available, or the test assumptions are not satisfied. In these scenarios, the permutation test is the default procedure to form the null distribution of the focal statistic (Berry et al. 2011; Chung and Romano 2013). Permutation tests have desirable properties that can guarantee exactness if data are exchangeable, and the procedure is very straightforward, so it can be applied to most test statistics. Consequently, they have become a popular tool among researchers in fields of genetics and other life sciences since they facilitate the design of novel statistics to ascertain statistical (Doerge and Churchill 1996; Nichols and Holmes 2002; Simpson et al. 2013). Nonetheless, it is important to recognize the drawbacks of permutation tests. They can be computationally expensive and time-consuming, rendering them impractical to generate all possible permutations in some scenarios.

In genetic studies involving large-scale omics data, the threshold for statistical significance is often set at very small *P*-value levels due to the multiple testing corrections (Benjamini and Hochberg 1995). For instance, to account for multiple testing in genome-wide association studies (GWAS), a stringent *P*-value threshold of $5\times 10^{-8}$ is commonly adopted to identify the association between a common genetic variant and a trait of interest (The International HapMap Consortium 2005; Pe’er et al. 2008). Likewise, gene-based analyses employ the Bonferroni correction to adjust the *P*-value threshold, typically calculated as 0.05 divided by the number of genes being tested (Cui and Churchill 2003; McDermaid et al. 2019). Given these standards, permutation tests can quickly become computationally demanding, especially when estimating exceedingly small *P*-values across a multitude of tests. Consequently, there is a pressing need for more efficient methods in such high-dimensional settings. Several recent studies have developed efficient algorithms based on permutation tests: Zhang et al. estimated genome-wide significance based on Poisson de-clumping heuristics (Zhang and Liu 2011); Segal et al. employed fast asymptotic approximation of small *P*-values by a partitioning and resampling scheme (Segal et al. 2018); and Yang et al. approximated small *P*-values using sequential Monte Carlo and the Edgeworth expansion (Yang et al. 2019). Despite these advancements, the scope of these methods remains confined to specific test statistic families. Our goal is to bridge this gap by introducing a versatile model-free protocol that applies to any test statistic without limiting its form.

The idea of this work is to replace the generation of vast numbers of permuted test statistics, by maximizing the utility of limited permutated statistics. Kernel-based density estimation (KDE) provides one such approach. KDE serves as a nonparametric way to estimate the probability density function (PDF) of a random variable (Scott et al. 1977; Jones and Kappenman 1992; Chen 2017; Silverman 2018). Even though KDE's foundations trace back to the pioneering work of Rosenblatt and Parzen over 50 yr ago (Rosenblatt 1971), continuous advancements in computing have expanded its application across a wide spectrum of scientific fields (Tsai and Chen 2007; King et al. 2016; Węglarczyk 2018). By applying a kernel function—like the Gaussian—to each data point and then aggregating them, KDE captures the inherent PDF of the data. This results in a refined and smooth curve that can estimate the probability of any value within the dataset. In KDE, the choice of the kernel function, represented as *K*, and the bandwidth, denoted by *h*, control the smoothness of a density estimate. Notably, when employing KDE to model permuted test statistics, setting the bandwidth to zero yields results parallel to a conventional Naïve permutation test (Epstein et al. 2012).

In our study, we introduce the Kernel-smoothed permutation method, leveraging KDE to model the null distribution of permuted test statistics, with the objective of refining the accuracy of small *P*-value estimations (eg $<10^{-6}$). The integration of a kernel function in KDE enables a broader representation of each permuted point in forming the null distribution's properties, reducing the number of permutations required. In scenarios with heavy-tailed test statistics that can hamper the efficacy of kernel smoothing, we developed a kurtosis-driven Box-Cox transformation, a step prior to the KDE application. Crucially, as a nonparametric method, Kernel-smoothed permutation does not require restrictive assumptions about parametric families. This flexibility permits its application across all spectrums of test statistics to construct a density that truly captures the inherent structure of the data.

In our framework, the standard *P*-value and its corresponding test statistic (X-standard), calculated from the closed-form distribution before permutation, serve as the gold standard for each test. We then performed the corresponding permutations following the specific procedures of each test and computed *P*-values using both the Naïve and the Kernel-smoothed permutation approaches, the latter incorporating KDE with different transformations. To evaluate the Kernel-smoothed permutation, we applied it to 3 test statistics with known closed-form distributions: the 2-sample *t*-test, the gene-based sequence kernel association test (SKAT) (Wu et al. 2010, 2011), and the chi-squared test. The Wellcome Trust Case Control Consortium (WTCCC) (Burton et al. 2007) data are used for comparison. Across these settings, side-by-side comparisons demonstrated that the Kernel-smoothed permutation consistently yielded more accurate results than the Naïve permutation when based on the same number of permuted test statistics. Notably, with a reduced (10%) set of test statistics of an order of magnitude, Kernel-smoothed permutation can still outperform Naïve permutation.

In the rest of this paper, we began by providing a comprehensive overview of the Kernel-smoothed permutation's underlying mechanics and rationales, followed by a detailed narrative on the methods behind our implementation of the Kernel-smoothed permutation technique. Moreover, in addition to presenting its applications to the 3 aforementioned tests, we also compared it with the existing efficient permutation method, fastPerm (Segal et al. 2018), in both the 2-sample *t*-test and the chi-squared test. We found that the Kernel-smoothed permutation, whether applied to full samples or 10% sub-samples, significantly outperforms the fastPerm method. Finally, in a real-world GWAS analysis using the WTCCC Crohn's disease (CD) cohort, the Kernel-smoothed permutation further demonstrated clear superiority over the Naïve permutation.

## Materials and methods

### Conceptual framework of the Kernel-smoothed permutation

**Fig. 1.**
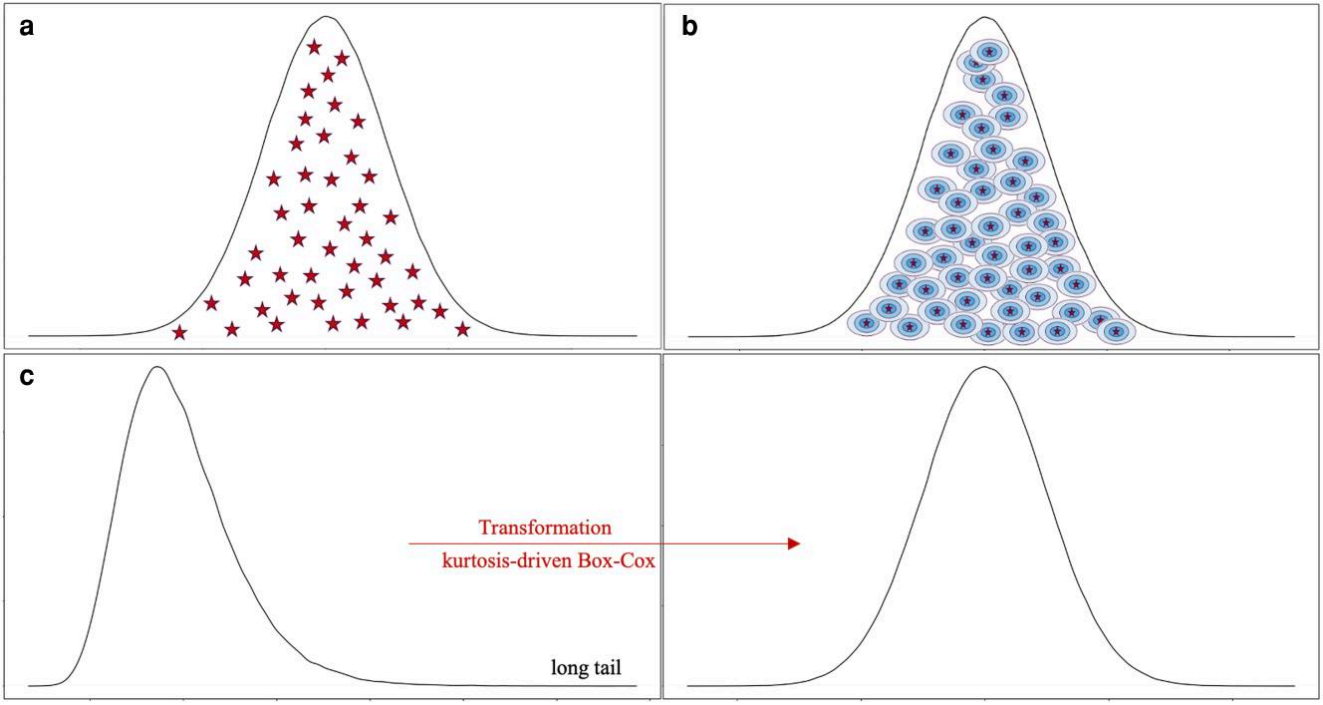
Overview of the Kernel-smoothed permutation protocol. a) In the Naïve permutation, each permuted sample is self-representative. b) In the Kernel-smoothed permutation, each permuted sample represents a broader region via the distribution centered by each sample. The kernel estimator learns the density as the sum of distributions placed at these samples. c) To estimate a very small *P*-value in genetic association studies, the heavy-tailed distribution will reduce the effectiveness of kernel smoothing. The kurtosis-driven Box-Cox transformation relieves the problem.

In the Naïve permutation, each permuted sample is self-representative. After generating a large set of these permuted samples, they are arranged in ascending order, allowing us to determine the rank of the test statistic (in the original hypothesis test) among these samples (Fig. 1a). Conversely, the Kernel-smoothed permutation allows each permuted sample to represent a broader region in the format of a kernel-based distribution (illustrated by the contours surrounding each sample in Fig. 1b). Here, the kernel function *K* determines their shapes, and the window *h* determines their bandwidth. The kernel-estimated null distribution is formed by aggregating these sample-centered distributions, leveraging extra information covered by the bandwidth of these kernels. Such a kernel smoothing can reduce the number of permutations required by exploiting each sample more thoroughly than a Naïve permutation (which is equivalent to a kernel with zero bandwidth). In genetic association studies, when computing a very small *P*-value, the heavy-tailed distribution can undermine the efficacy of kernel smoothing, which mandates a transformation of samples. We have then developed a kurtosis-driven Box-Cox transformation that narrows down the relative excess kurtosis toward a normal distribution (Fig. 1c).

### Univariate Kernel-based density estimation

Given *n* data points $X_{1},\ldots ,X_{n}$ drawn from an independent and identically distributed (*iid*) sample from a population *X* with an unknown continuous PDF f(x), the KDE is defined as:


$$\hat{\;f}(x,h)=\frac{1}{nh}\overset{n}{\underset{i=1}{\sum }}\;K\left(\frac{x-X_{i}}{h}\right)$$

Here, *K* represents the kernel function, and *h* is the smoothing parameter or bandwidth. In our subsequent KDE analysis, we focused on the most used Gaussian kernel with kernel function


$$K(x)=\frac{1}{\sqrt{2\pi }}e^{\frac{-x^{2}}{2}}$$

The choice of the smoothing parameter, also known as the bandwidth *h*, is pivotal, as it dictates the extent of smoothing applied. A very small value of *h* can lead to many wiggly structures on the kernel estimate, and this is a signature of under-smoothing, where the amount of smoothing is too small so some structures identified by KDE might be caused by randomness. Conversely, when *h* is too large, the computed density will be over-smoothed, where some important structures are obscured by the huge amount of smoothing, but its variance across different samples is reduced.

The performance of KDE is measured by MISE (mean integrated squared error) or AMISE (asymptotic mean integrated squared error). The core idea behind different bandwidth selection methods is to minimize the AMISE, and different methods can be viewed as different estimators to minimize the AMISE. There are several commonly used methods to select the bandwidth in KDE (Bowman 1984; Scott and Terrell 1987; Jones et al. 1996; Scott 2011; Silverman 2018), in our subsequent analyses, we set the bandwidth to Silverman's rule-of-thumb bandwidth (Silverman 2018) multiplied by a factor ranging from 1 to 15.

### Closed-form tests applied in our analysis

Our Kernel-smoothed permutation protocol is versatile and can be adapted to any distribution-free test statistic. To validate its efficacy, we applied it to 3 established closed-form tests: the 2-sample *t*-test, the SKAT (Wu et al. 2010, 2011), and the chi-squared test.

#### Two-sample *t*-test

We aim to test whether there is a significant difference between 2 groups, A and B, within a population assuming equal variances. Under the null hypothesis, the test statistic *T* follows the t-distribution and the corresponding *P*-value is derived as $P(T^{2}>T_{0}^{2})$. The degrees of freedom of the t-distribution are given by $df=n_{A}+n_{B}-2$, where $n_{A}$ is the sample size of Group A, and $n_{B}$ is the sample size of Group B.

#### Sequence kernel association test

SKAT is a region-based association method designed to evaluate the joint effects of multiple genetic variants within a particular region on a phenotype, while adjusting for covariates such as principal components to control for population stratification. It employs a multiple regression framework that models the phenotype as a function of the genetic variants in the region and relevant covariates, allowing each variant to have potentially different effect directions and magnitudes, including null effects. Instead of estimating individual regression coefficients, SKAT applies a variance-component score test within a mixed-model framework to assess whether the combined effect of the variants is significantly associated with the phenotype.

Supposing there are *p* variants with *n* subjects sequenced in a region. Let *y* denotes the phenotype variable, *X* be the covariates matrix, and *G* stand for the genotype matrix for the *p* variants in the region. We then consider a logistic regression model when the phenotypes are dichotomous (binary) traits:


$$logit\;P(y=1)=\alpha_{0}+\alpha^{\prime }X+\beta^{\prime }G,$$

Here, $\alpha_{0}$ is an intercept term, *α* is the vector of regression coefficients for the covariates, *β* is the vector of regression coefficients for the *p* observed genetic variants in the region. The corresponding null hypothesis is: $H_{0}:\;\beta =0$. To increase the power, SKAT tests $H_{0}$ by assuming each $\beta_{j}$ follows an arbitrary distribution with a mean of zero and a variance of $w_{j}\tau$, where *τ* is a variance component and $w_{j}$ is a pre-specified weight for variant *j*. Finally, the null hypothesis changes to $H_{0}:\;\tau =0$, which can be tested using a variance-component score test:


$$Q=(y-\hat{\mu }{)}^{\prime }K(y-\hat{\mu })$$

where $K=GWG^{\prime }$, $\hat{\mu }$ is the predicted mean of *y* under $H_{0}$, $W=diag(w_{1},\ldots ,w_{p})$ contains the weights of the *p* variants. Under the null hypothesis, *Q* follows a mixture of chi-square distributions, which can be closely approximated with the computationally efficient Davies method (Davies 1980).

#### Chi-squared test

We aim to test for association between genetic information and binary traits (case and control status) in GWAS studies using the chi-squared test. The *P*-value is then derived using the chi-squared distribution as $P(\chi^{2}>\chi_{0}^{2})$. The degrees of freedom of the chi-squared distribution are given by df=(r−1)(c−1)=(3−1)(2−1)=2, where *r* denotes the number of rows, and *c* denotes the number of columns in the contingency table.

### Data sources and quality control

For the 2-sample *t*-test, both groups were assigned the same sample size, which was randomly selected from the set: 100, 150, 200, 250, and 500. The population mean of the first group was derived from a Uniform distribution U(0,1), while the population mean for the second group was fixed at 0. The population variance, which was identical for both groups, was chosen from the set {1,2,3,4,5}. Subsequently, samples for both groups were drawn from a normal distribution, using the aforementioned parameters. In R, the built-in function **t.test** function was used to perform the 2-sample t-test, with the confidence level set to 0.95.

In SKAT, the genotype information is from the representative GWAS dataset, the WTCCC. For our analysis, we selected rheumatoid arthritis (RA) samples, comprising 4,798 individuals (1,860 cases and 2,938 controls). We excluded genetic variants with a minor allele frequency (MAF) of 1% or less, resulting in a pruned set of 392,937 variants for our subsequent analysis. The gene annotation for the human genome is from GENCODE, retaining 19,096 ENSEMBL genes for further study. The test was conducted using the **SKAT** function from the **SKAT** package in R. Detailed observed and expected genotype counts for cases and controls in the WTCCC RA dataset are provided in Supplementary Table 22.

For the chi-squared test, we sourced both genetic and phenotype data from the RA samples in the WTCCC, consistent with our SKAT analysis. In R, we used the built-in **chisq.test** function to execute the chi-squared test.

### Naïve permutation *P*-value calculation

For each round of permutation, we randomly switched labels and calculated the associated test statistic. Let *M* represents the number of permutation iterations. After *M* permutations, we obtained $X_{1},\ldots ,X_{M}$ as the permuted test statistics. The *P*-value from the Naïve permutation is given by:


$$P_{\mathrm{Na}\ddot{\text{ı}}\mathrm{ve}\;}=\frac{\#\;(X_{i}\geq X_{standard})}{M}$$

for 1≤i≤M. Here, #() denotes the number of times the event is satisfied. $X_{standard}$ represents the test statistic calculated from the above closed-form tests.

In the 2-sample *t*-test, we initially combined 2 groups to form a sample of $N_{A}+N_{B}$ data points. Then, we draw, without replacement, $N_{A}$ points from the $N_{A}+N_{B}$ points to form group A, with the remaining $N_{B}$ points forming group B. For both the SKAT and chi-squared tests, we shuffled the “case” and “control” labels among the 4,798 RA individuals. Afterward, we computed the permuted test statistics and determined the Naïve permutation *P*-value for all 3 tests using the above equation.

### Transformations considered for permuted test statistics

Permuted test statistics derived from both the SKAT and the chi-squared test exhibit a heavy-tailed distribution, which can diminish the efficacy of KDE. To address this, we explored transforming these permuted test statistics. Our analysis considered 7 distinct transformations:

1. Original transformation: retain the permuted test statistics in their original state, use $X=(X_{1},\ldots ,X_{M})$ as permuted test statistics in kernel-smoothing.
2. Squared root transformation: use $X^{1/2}$ as permuted test statistics in kernel-smoothing.
3. Cubic root transformation: use $X^{1/3}$ as permuted test statistics in kernel-smoothing.
4. Log transformation: use ln(X) as permuted test statistics in kernel-smoothing.
5. Box-Cox transformation: The Box-Cox transformation is a statistical method that transforms non-normal data into an approximately normal distribution. The transformation function is given for different values of the transformation parameter *λ* by the following expression.
  $$\{\begin{array}{c} \frac{X^{\lambda }-1}{\lambda }\;if\;\lambda \neq 0\\ \log\;(X)\;if\;\lambda =0\; \end{array}$$

In R, we have the **boxcox** function from the **MASS** package to estimate the optimal *λ* by maximum likelihood estimation.

6 and 7. Skewness-driven and kurtosis-driven Box-Cox transformation: While the Box-Cox transformation is designed to make data resemble a normal distribution, the results might not always exhibit the ideal skewness and kurtosis inherent to the normal distribution (a skewness of 0 and a kurtosis of 3). To address this, we introduce 2 additional transformations: the skewness-driven and kurtosis-driven Box-Cox transformations. The skewness-driven Box-Cox transformation optimizes the parameter λ by minimizing the absolute value of skewness in the transformed data. Conversely, the kurtosis-driven Box-Cox transformation optimizes λ to minimize the relative excess kurtosis toward a normal distribution, defined as the absolute difference between the sample kurtosis and 3. These tailored transformations allow for more nuanced adjustments, targeting specific moments of the distribution to better align with the characteristics of the normal distribution.

### Analysis procedures and settings in the Kernel-smoothed permutation protocol

Our protocol is a 5-step implementation. We initiated the process with hypothesis testing for each closed-form test and obtained the corresponding *P*-value, which we referred to as the standard *P*-value. Throughout this study, the test statistic corresponding to the standard *P*-value, denoted as X-standard, together with the standard *P*-value itself, serves as the gold standard for comparison. Given computational restrictions, we opted for standard *P*-value accuracy thresholds of $10^{-7}$ and $10^{-8}$. Next, using the standard *P*-value accuracy threshold of $10^{-8}$ as an example, we generated $10^{8}$ permuted test statistics following the procedures in *Naïve permutation P-value calculation* and then calculated the Naïve permutation *P*-value. Third, we transformed the aforementioned $10^{8}$ permuted test statistics using the 7 transformations previously described. We termed them as full samples. Fourth, we implemented the KDE on the full samples utilizing the **kde** function from the **utilities** package in R. This function outputs a KDE object encompassing different probability functions derived from the KDE process. To compute the Kernel-smoothed permutation *P*-value, we employed the **pkde** function (R package **utilities**) from the resulting object. To evaluate the effectiveness of our method across different bandwidth lengths, we adjusted Silverman's rule-of-thumb bandwidth by multiplying it with scaling factors ranging from 1 to 15 in increments of 0.5. Our objective here was to identify the optimal bandwidth coefficient and the most effective transformation, ensuring the Kernel-smoothed permutation *P*-value aligns closest to the standard *P*-value. Finally, we repeated the procedures described in the fourth step but applied them to a subset of $10^{8}$ transformed test statistics, which we termed 50% and 10% sub-samples.

For the 2-sample *t*-test, we evaluated our protocol 100 times under 2 *P*-value accuracy thresholds, ie applying the Kernel-smoothed permutation in 100 different settings for each threshold. Within each setting, we repeated the procedure 50 times and computed both Naïve permutation and Kernel-smoothed permutation *P*-values. The median of these 50 replicates was taken as the final *P*-value for each method. We chose the median because it is more robust to extreme values, particularly when the *P*-value distribution is right-skewed. For SKAT, we applied the protocol to 4 significant genes (2 genes per accuracy threshold). For each gene, the procedure was repeated 50 times, and the final *P*-values for Naïve and Kernel-smoothed permutations were obtained as the medians of the 50 replicates. For the chi-squared test, we applied the protocol to 34 significant genetic variants under 2 accuracy thresholds. Similarly, each genetic variant was analyzed with 50 replicates, and the final *P*-values were taken as the medians of the replicated values.

### Comparison of *P*-values between the kernel-smoothed and naïve permutations

To compare the effectiveness of *P*-values derived from the Kernel-smoothed permutation and the Naïve permutation relative to the standard *P*-value, we utilized the -log_10_(*P*-value) metric. Specifically, for both Kernel-smoothed and Naïve permutation *P*-values, we firstly calculated the -log_10_() of the values. Subsequently, we established their deviation from the standard *P*-value (also expressed in -log_10_() terms) by taking the absolute difference. Our protocol, the Kernel-smoothed permutation, is deemed superior when its absolute -log_10_(*P*-value) difference is less than that of the Naïve permutation.

### Comparison with the fastPerm method and significance testing

To further evaluate the performance of the Kernel-smoothed permutation, we also compared it with an established efficient permutation approach. Specifically, we adopted the method proposed by Segal et al., namely an asymptotic approximation combined with a resampling algorithm for rapidly estimating small permutation *P*-values for the difference and ratio of means in 2-sample tests^13^ (hereafter referred to as the fastPerm method). Implemented in the R package **fastPerm**, this approach was applied to the same 2-sample *t*-test and chi-squared test datasets as the Kernel-smoothed permutation, yielding the corresponding fastPerm *P*-values under the accuracy thresholds of $10^{-7}$ and $10^{-8}$. In addition, we incorporated the Wilcoxon signed-rank test to examine whether the differences among the 3 permutation methods were statistically significant.

### Application of Kernel-smoothed permutation in GWAS of the Crohn's disease cohort

To evaluate the Kernel-smoothed permutation in practice, we benchmarked its application in a GWAS of the WTCCC CD cohort, which included 1,748 cases and 2,938 controls. Genotype data were processed using PLINK 1.9 with standard quality control (QC) procedures, including the removal of markers with a missingness rate greater than 0.1, MAF below 0.05, and significant deviations from Hardy–Weinberg equilibrium (*P* < 1× 10^−6^). Samples with a missingness rate above 0.1 were also excluded. After QC, the dataset comprised 4,686 individuals and 348,814 SNPs for analysis.

Since the CD phenotype is dichotomous, GWAS was performed using logistic regression under an additive genetic model in PLINK 1.9 to obtain the standard *P*-value for each SNP. The likelihood ratio test was used for association testing, corresponding to a chi-square test with one degree of freedom. At the genome-wide significance threshold of $5\times 10^{-8}$, 19 significant SNPs were identified; however, due to computational considerations, only ten of these SNPs were selected for subsequent comparative analyses between the Naïve permutation and Kernel-smoothed permutation methods (Supplementary Table 17). At the threshold of $5\times 10^{-7}$, 14 additional significant SNPs were identified, all of which were included in the subsequent analyses (Supplementary Table 18). The observed and expected genotype counts for cases and controls in the WTCCC CD dataset are provided in Supplementary Table 22.

## Results

### Kernel-smoothed permutation exhibits superior performance over naïve permutation

**Fig. 2.**
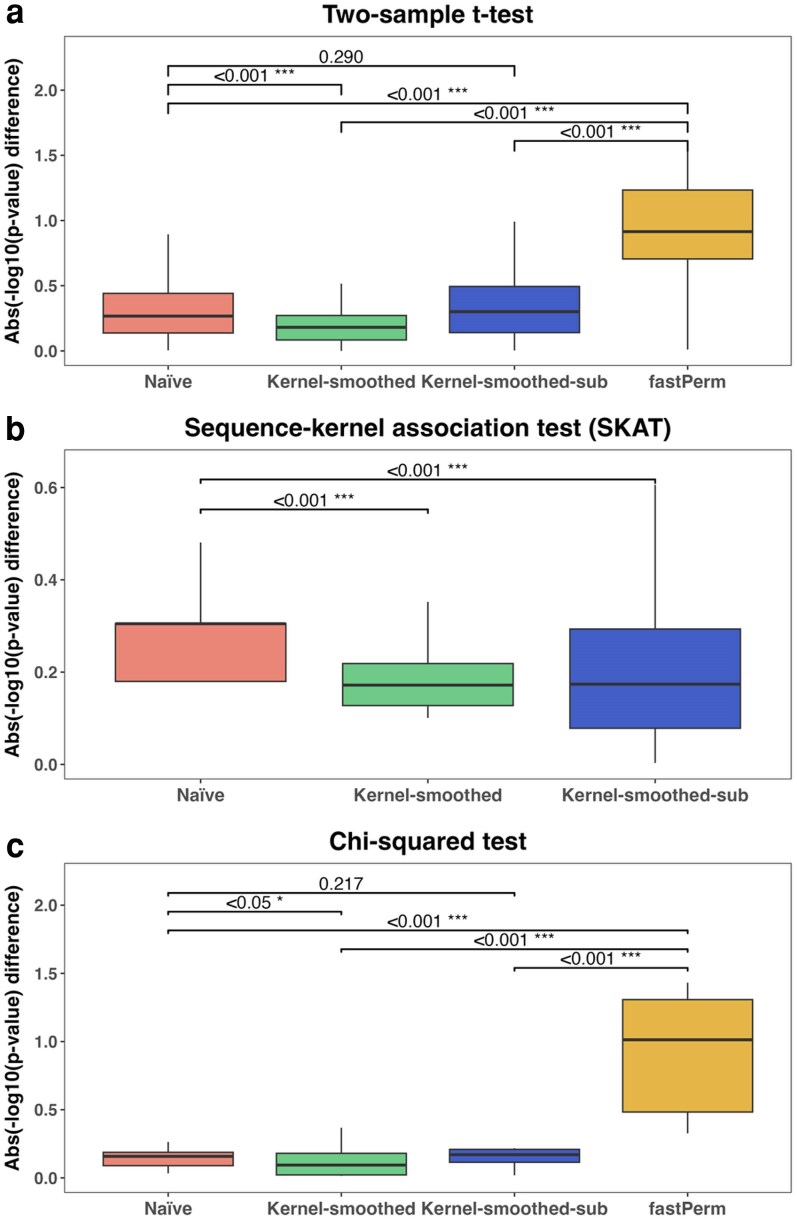
Comparison between the Naïve permutation (red), Kernel-smoothed permutation (green for full samples and blue for 10% subset samples) under the optimal transformation, and fastPerm method (yellow). *P*-values are compared in absolute -log_10_(*P*-value) difference for a *P*-value accuracy threshold of $10^{-8}$ and an optimal bandwidth coefficient of 7. a) represents the 2-sample *t*-test, with the optimal transformation as original (no transformation); b) represents the SKAT (gene *IP6K3*), with the optimal transformation as log transformation; and (c) represents the chi-squared test, with the optimal transformation as kurtosis-driven Box-Cox transformation.

We firstly implemented our method on a 2-sample *t*-test across 2 *P*-value accuracy thresholds based on different configurations described in MATERIALS AND METHODS. At a *P*-value accuracy threshold of $10^{-8}$, the Kernel-smoothed permutation outperformed Naïve permutation utilizing $10^{8}$ original (non-transformed) permuted full samples, when the optimal bandwidth coefficient was set to 7. The paired Wilcoxon signed-rank test confirmed a significant difference between the 2 methods (*P* < 0.001). Although when only 10% sub-samples were used, the improvement of the Kernel-smoothed permutation relative to the naïve permutation was limited (*P* = 0.290) (Fig. 2a). At a *P*-value accuracy threshold of $10^{-7}$, the Kernel-smoothed permutation steadily outperformed Naïve permutation with an optimal bandwidth coefficient of 5, based on both $10^{8}$ original (non-transformed) permuted full samples and only 10% sub-samples (*P* < 0.001) (Supplementary Fig. 1a). These 2 optimal bandwidth coefficients are consistent with the outcomes observed in the other 2 closed-form tests within the same *P*-value accuracy threshold.

Secondly, after running SKAT on RA samples from the WTCCC dataset, we identified 4 and 15 significant genes at *P*-value accuracy thresholds of $10^{-7}$ and $10^{-8}$, respectively. Due to computational constraints, we restricted our application of the Kernel-smoothed permutation in SKAT to 4 genes across 2 *P*-value accuracy thresholds, following the configurations described in MATERIALS AND METHODS. Specifically, we applied Kernel-smoothed permutation to the genes *UQCC2* and *IP6K3* for a *P*-value accuracy threshold of $10^{-8}$, and to the genes *PPP1R18* and *LEMD2* for a *P*-value accuracy threshold of $10^{-7}$. In our figure illustration, we have chosen to display the results for genes *IP6K3* and *LEMD2*. However, detailed results of these 4 genes can be found in Supplementary Tables 1–4. For *IP6K3*, the Kernel-smoothed permutation outperformed Naïve permutation utilizing both $10^{8}$ log-transformed full samples and 10% sub-samples with a paired Wilcoxon signed-rank test indicating a significant difference between the 2 methods (*P* < 0.001) (Fig. 2b). For *LEMD2*, the Kernel-smoothed permutation significantly outperformed the Naïve permutation when utilizing $10^{7}$ log-transformed full samples (*P* < 0.001). When only 10% of the samples were used, the advantage of the Kernel-smoothed permutation over the Naïve permutation was not substantial (*P* = 0.186) (Supplementary Fig. 1b).

Finally, after performing the chi-squared test on RA samples from the WTCCC dataset, we identified 18 and 16 significant genetic variants at *P*-value accuracy thresholds of $10^{-7}$ and $10^{-8}$, respectively. We implemented our method on these significant variants across 2 *P*-value accuracy thresholds, following the configurations described in MATERIALS AND METHODS. For both *P*-value accuracy thresholds, the Kernel-smoothed permutation outperformed Naïve permutation using $10^{8}$ kurtosis-driven Box-Cox transformed full samples, with a paired Wilcoxon signed-rank test indicating a significant difference (*P* < 0.05). When restricted to 10% sub-samples, the Kernel-smoothed permutation showed no clear advantage over the Naïve permutation (*P* = 0.217 and *P* = 0.495) (Fig. 2c; Supplementary Fig. 1c).

It is worth noting that, under both the 2-sample *t*-test and the chi-squared test, the fastPerm method consistently performed the worst when compared with the Kernel-smoothed permutation (using either full samples or 10% sub-samples) and the Naïve permutation. The paired Wilcoxon signed-rank test confirmed that their differences were highly significant (*P* < 0.001) (Fig. 2a and c; Supplementary Fig. 1a and c; Supplementary Tables 13–16). The fastPerm *P*-values exhibited an upward bias compared with the standard *P*-values, with both their bias and variance substantially exceeding those of the Naïve and Kernel-smoothed permutation methods (Supplementary Table 23).

### Kurtosis-driven Box-Cox transformed samples perform best in the Kernel-smoothed application

It is noteworthy that permuted samples derived from the 2-sample *t*-test follow the studentized t-distribution. As the sample size increases, this t-distribution approaches the normal distribution. Given this behavior, it is justifiable to retain the permuted samples in their original state, especially because their skewness and kurtosis already closely mirror the attributes of a normal distribution.

**Fig. 3.**
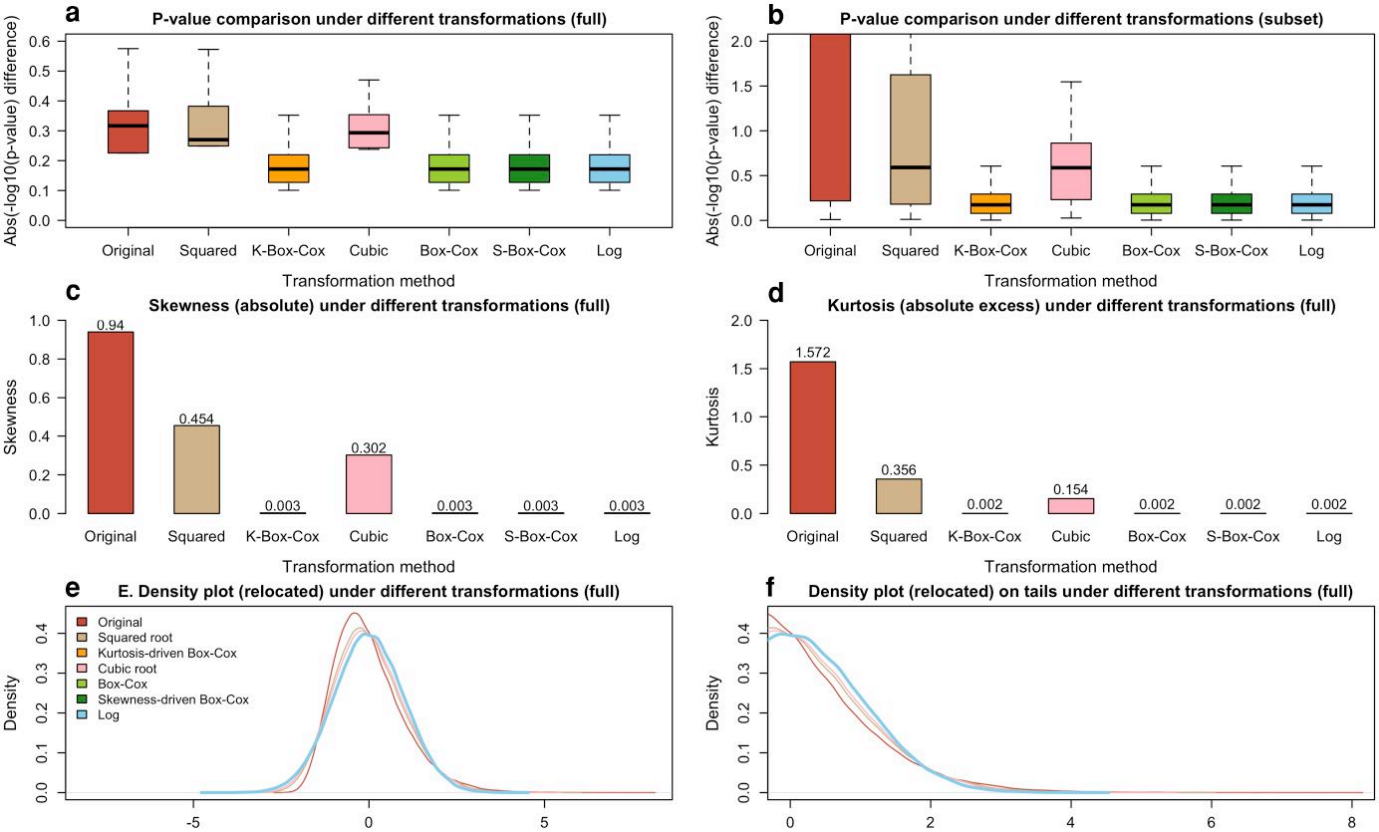
Comparison of *P*-values from Kernel-smoothed permutation under different transformations in SKAT (gene *IP6K3*). *P*-values are compared in absolute -log_10_(*P*-value) difference for a *P*-value accuracy threshold of $10^{-8}$ and an optimal bandwidth coefficient of 7. a) represents the *P*-value comparison for full samples; b) represents the *P*-value comparison for 10% sub-samples; c) and (d) represent the skewness and kurtosis values under different transformations for full samples; and (e) and (f) represent the density (relocated) plots underlying distributions of various transformations along with their focused visuals of the tails for full samples.

The permuted samples generated from SKAT follow a mixture of chi-squared distributions and are heavy-tailed (Fig. 3e and f; Supplementary Fig. 2e and f). Employing a log transformation effectively converts these samples into a symmetric distribution characterized by optimal skewness and kurtosis aligning with a normal distribution. We also presented a *P*-value comparison under different transformations for both full samples and 10% sub-samples (Fig. 3a and b; Supplementary Fig. 2a and b). For gene *IP6K3*, log transformation performed the best among all transformations using both full samples and 10% sub-samples. For gene *LEMD2*, log transformation performed the best using full samples but performed almost the same as squared root transformation (still outperformed the others) using 10% sub-samples. When considering the full samples, we also assessed their skewness and kurtosis under different transformations (Fig. 3c and d; Supplementary Fig. 2c and d). The efficacy of the Kernel-smoothed permutation across various transformations seemed consistent with their respective skewness and kurtosis values. Moreover, the underlying distributions of various transformations are illustrated through their density plots, accompanied by focused visuals of the tails (Fig. 3e and f; Supplementary Fig. 2e and f).

Notably, within SKAT, the **boxcox** function estimated the optimal λ value at 0, indicating a preference for log transformation. Given that the log-transformed permuted samples already exhibit optimal skewness and kurtosis properties, there is no need to perform the 3 Box-Cox transformations, as they are the same in principle. Nevertheless, for the sake of visual consistency, we incorporated results under the 3 Box-Cox transformations in Fig. 3, where their performance mirrored that of the log transformation.

**Fig. 4.**
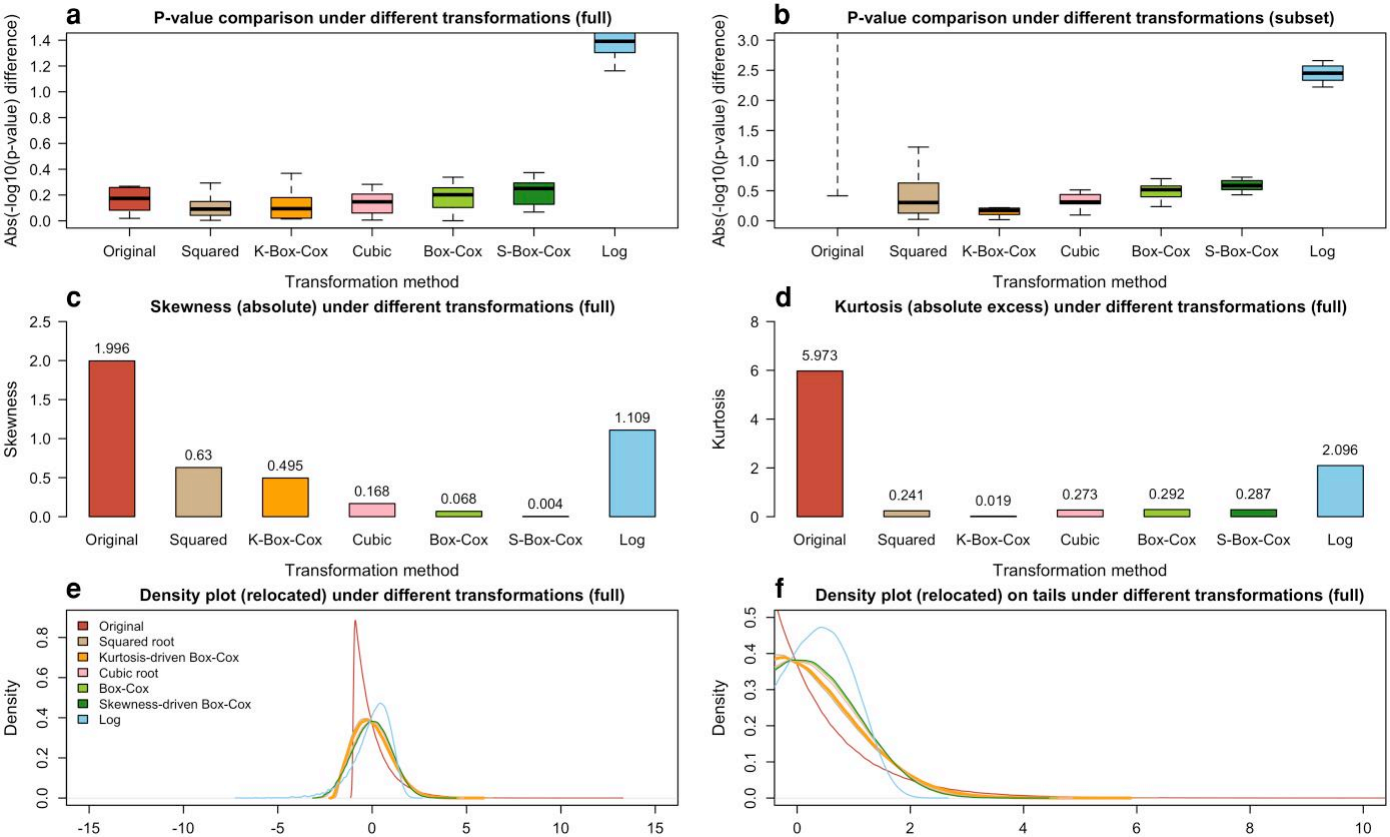
Comparison of *P*-values from Kernel-smoothed permutation under different transformations in the chi-squared test. *P*-values are compared in absolute -log_10_(*P*-value) difference for a *P*-value accuracy threshold of $10^{-8}$ and an optimal bandwidth coefficient of 7. a) represents the *P*-value comparison for full samples; b) represents the *P*-value comparison for 10% sub-samples; c) and (d) represent the skewness and kurtosis values under different transformations for full samples; and (e) and (f) represent the density (relocated) plots underlying distributions of various transformations along with their focused visuals of the tails for full samples.

The permuted samples generated from the chi-squared test follow a chi-squared distribution and are heavy-tailed (Fig. 4e and f; Supplementary Fig. 3e and f). Employing a kurtosis-driven Box-Cox transformation effectively converts these samples into a symmetric distribution characterized by optimal kurtosis aligning with a normal distribution. We also presented a *P*-value comparison under different transformations for both full samples and 10% sub-samples (Fig. 4a and b; Supplementary Fig. 3a and b). For a *P*-value accuracy threshold of $10^{-8}$, the kurtosis-driven Box–Cox transformation achieved the second-best performance among all transformations when using the full samples, following the squared root transformation. Whereas in 10% sub-samples, the kurtosis-driven Box–Cox transformation yielded the best performance. For a *P*-value accuracy threshold of $10^{-7}$, kurtosis-driven Box-Cox transformation did not outperform the squared root transformation but still outperformed the others using full and 10% sub-samples. Remarkably, during the chi-squared test, the **boxcox** function determined that the optimal λ value for the kurtosis-driven Box-Cox transformation was approximately 0.45. This value is quite close to the transformation parameter of 0.5 typically employed in the square root transformation. Consequently, this similarity in transformation parameters partly accounts for the occasional lack of significant improvement observed with the kurtosis-driven Box-Cox transformation when compared to the square root transformation. When considering the full samples, we also assessed their skewness and kurtosis under different transformations (Fig. 4c and d; Supplementary Fig. 3c and d). The efficacy of the Kernel-smoothed permutation across various transformations seemed almost consistent with their respective skewness and kurtosis values. Moreover, the underlying distributions of various transformations are illustrated through their density plots, accompanied by focused visuals of the tails (Fig. 4e and f; Supplementary Fig. 3e and f). Detailed information on the above results can be found in Supplementary Tables 5–12.

### Strong performance of the Kernel-smoothed permutation in GWAS of the Crohn's disease cohort

**Fig. 5.**
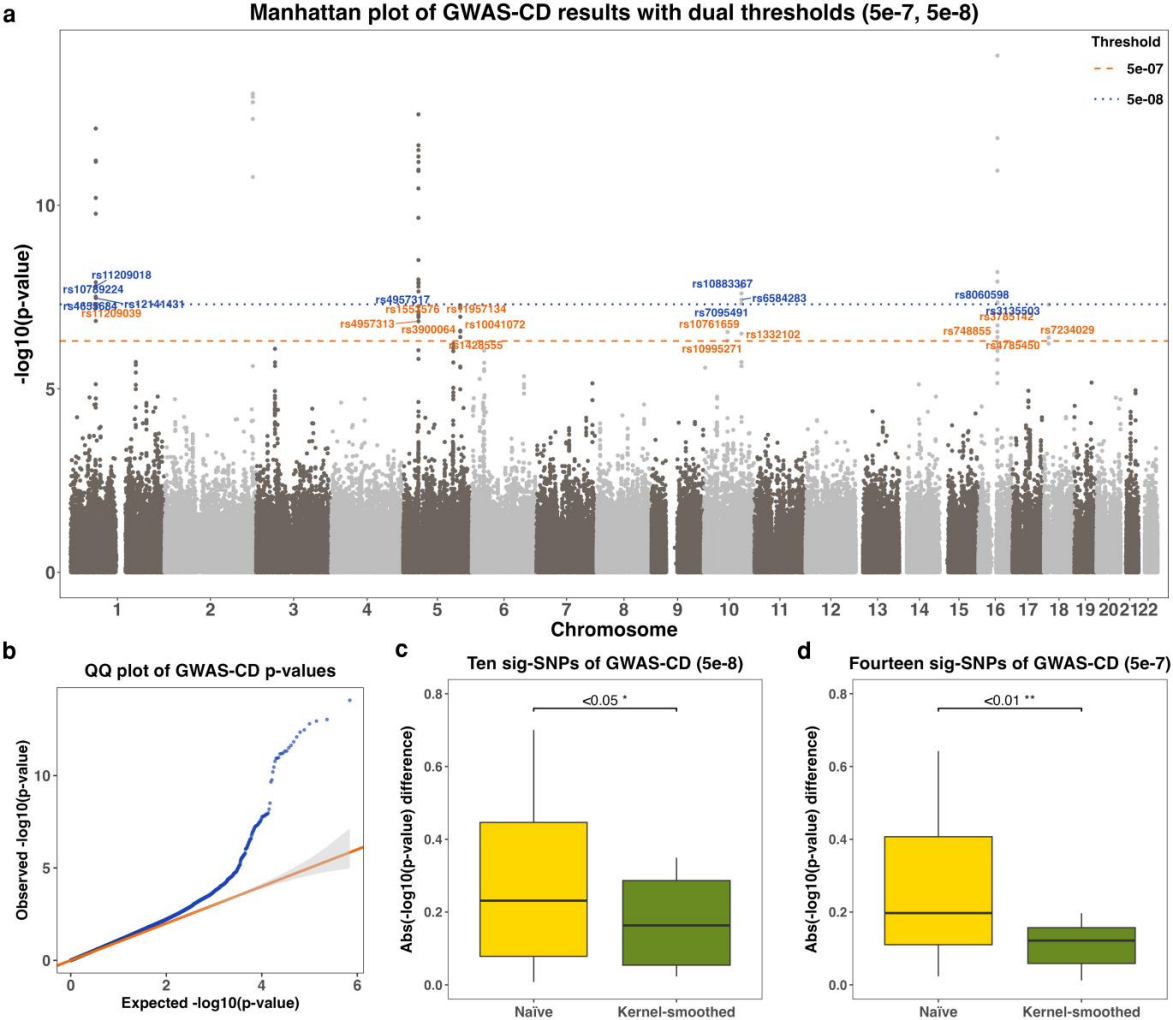
Comparison of *P*-values from Naïve and Kernel-smoothed permutations in GWAS-CD results. *P*-values are compared in absolute -log_10_(*P*-value) difference at accuracy thresholds of $5\times 10^{-7}$ and $5\times 10^{-8}$. a) Manhattan plot of GWAS-CD results; b) QQ plot of GWAS-CD results; c) comparison of *P*-values for 10 significant genetic variants identified in GWAS-CD at a threshold of $5\times 10^{-8}$; d) comparison of *P*-values for 14 additional significant genetic variants identified in GWAS-CD at a threshold of $5\times 10^{-7}$. The GWAS-CD analysis was conducted using logistic regression under an additive genetic model, with association testing performed using the likelihood ratio test, which follows a chi-square distribution with one degree of freedom.

After performing a standard GWAS analysis of CD (GWAS-CD) using PLINK 1.9, we selected 10 significant SNPs at the threshold of $5\times 10^{-8}$ and 14 additional significant SNPs at the threshold of $5\times 10^{-7}$ for comparative analyses of the Naïve and Kernel-smoothed permutation methods (Supplementary Tables 17 and S18). The Manhattan plot (Fig. 5a) and QQ plot (Fig. 5b) summarize the GWAS-CD results and highlight the 24 selected significant SNPs. At the threshold of $5\times 10^{-8}$, the 10 significant SNPs were each evaluated with no fewer than $10^{8}$ permutation tests. The resulting Z-statistics were then used to calculate Naïve and Kernel-smoothed permutation *P*-values. As shown in Fig. 5c, the Kernel-smoothed permutation consistently outperformed the Naïve permutation, and the paired Wilcoxon signed-rank test confirmed a significant difference between the 2 methods (*P* < 0.05). Similarly, for the 14 additional significant SNPs at the $5\times 10^{-7}$ threshold, each SNP was subjected to at least $10^{7}$ permutation tests to obtain the corresponding Z-statistics, from which Naïve and Kernel-smoothed permutation *P*-values were derived. We found that the Kernel-smoothed permutation substantially outperformed the Naïve permutation (Fig. 5d), with a paired Wilcoxon signed-rank test indicating a significant difference between the 2 methods (*P <* 0.01).

## Discussion

In conclusion, we introduced the Kernel-smoothed permutation, a technique that utilizes kurtosis-driven Box-Cox transformation-guided KDE to model the null distribution of permuted test statistics, enhancing the precision of small *P*-value calculations. Our approach was tested across 3 closed-form tests, and consistently demonstrated superiority over the Naïve permutation in terms of *P*-value accuracy and efficiency in both full samples and 10% sub-samples, reducing the number of permutations needed. Our method was also compared with the fastPerm method, an existing efficient permutation scheme, in 2 closed-form tests, where it significantly outperformed fastPerm using both full samples and 10% sub-samples. The fastPerm *P*-values exhibited an upward bias relative to the standard *P*-values, and both their bias and variance were substantially larger than those of the Kernel-smoothed permutation (Supplementary Table 23). Furthermore, in a real-world GWAS-CD analysis, the Kernel-smoothed permutation demonstrated additional superiority over the Naïve permutation. As a nonparametric strategy, the Kernel-smoothed permutation does not hinge on any parametric prerequisites and can be seamlessly incorporated with any distribution-free test statistics. We anticipate that our methodology will be similarly effective for a broader spectrum of test statistics.

Our study presents certain limitations that merit consideration. Undoubtedly, the method of bandwidth selection has a substantial impact on the performance of the Kernel-smoothed permutation. In this study, we adopted Silverman's rule-of-thumb and explored candidate bandwidths ranging from 1 to 15 times the Silverman estimate to identify the optimal parameter. Under the 2 significance thresholds, although we attempted to balance the performance of the Kernel-smoothed permutation across the 3 closed-form tests when selecting the optimal bandwidth, this approach has inherent limitations: a single fixed bandwidth cannot guarantee optimal performance for all 3 tests simultaneously. For example, in the 2-sample *t*-test at the $10^{-7}$ threshold, using the full samples as well as 50% and 10% subsets, the Kernel-smoothed permutation with bandwidth = 5 outperformed both the Naïve permutation and the Kernel-smoothed permutations with bandwidths of 3 and 9 (Supplementary Fig. 4a–c; Supplementary Table 20). In contrast, at the $10^{-8}$ threshold, using the full samples, 50% and 10% subsets, the Kernel-smoothed permutation with bandwidth = 7 outperformed the Naïve permutation and the Kernel-smoothed permutation with bandwidth = 3, but it could not be consistently shown to outperform bandwidth = 9 (Supplementary Fig. 4d–f; Supplementary Table 21). These findings suggest that future improvements should consider more flexible, data-dependent bandwidth selection strategies tailored to different GWAS datasets and complex traits, or explore alternative bandwidth selection methods to further enhance the performance of the Kernel-smoothed permutation.

Using real-world genotype and phenotype data from the WTCCC CD cohort, we conducted a standard GWAS analysis. Based on the GWAS-CD results, we selected 10 significant SNPs at the threshold of $5\times 10^{-8}$ and 14 additional significant SNPs at the threshold of $5\times 10^{-7}$ for comparative analyses of the Naïve permutation and Kernel-smoothed permutation methods (Supplementary Tables 17 and 18). We observed that the Naïve permutation tests tended to underestimate the *P*-values of truly associated SNPs in 90.00 and 93.75% of cases, respectively; that is, the Naïve permutation *P*-values were smaller than the corresponding standard *P*-values. In contrast, the Kernel-smoothed permutation did not show a consistent tendency toward smaller *P*-values, with proportions of 60.00 and 42.86%, respectively, indicating no clear systematic bias. Moreover, the *P*-values obtained from the Kernel-smoothed permutation remained within the predefined thresholds, ensuring that the identification of significant SNPs was not compromised. To further evaluate whether these differences are driven primarily by bias or variance, we summarized the bias and variance of the permutation *P*-values (Supplementary Table 23). For Supplementary Tables 17 and 18, the mean Naïve permutation *P*-values were substantially lower than the mean standard *P*-values relative to the variance, indicating a systematic downward bias. This pattern was not observed for the Kernel-smoothed permutation, suggesting that the kernel-smoothed approach mitigates this bias more effectively than the Naïve permutation test. Similar trends were observed in the WTCCC RA data analysis. Taking the threshold of $10^{-8}$ as an example, the Naïve permutation *P*-values showed a higher proportion of values smaller than the standard *P*-values (75.00% in Supplementary Tables 5 and 6). However, for the Kernel-smoothed permutation with the kurtosis-driven Box–Cox transformation, the permutation *P*-values were not consistently smaller than the standard *P*-values (50.00% in Supplementary Table 5 and 56.25% in Supplementary Table 6). Overall, the Kernel-smoothed permutation, which incorporates smoothing and transformation steps, provides empirically less biased *P*-value estimates than the Naïve permutation test.

As a further comparison, we evaluated the computational time of the Naïve and Kernel-smoothed permutation methods after the permutation test statistics had already been generated. On average, the Kernel-smoothed permutation required roughly 3 min per SNP at the threshold of $5\times 10^{-7}$ and about 30 min per SNP at the threshold of $5\times 10^{-8}$, whereas the Naïve permutation took less than 1 min (Supplementary Fig. 5, Supplementary Table 19). By contrast, under the same computational settings, generating the permutation test statistics alone required approximately 24 h for $10^{7}$ replicates and 240 h for $10^{8}$ replicates because the statistical test must be recomputed for each permutation of the phenotype across all SNPs—several orders of magnitude longer than the runtime of the Kernel-smoothed permutation. Although the smoothing and transformation steps of the Kernel-smoothed permutation introduced additional computational overhead, this cost is negligible relative to the overall burden of permutation testing in GWAS. In future work, we will further optimize the Kernel-smoothed permutation protocol to mitigate *P*-value underestimation, explore alternative transformation strategies, and develop correction approaches that balance computational efficiency with statistical power.

In addition to the points discussed above, some additional limitations deserve more in-depth consideration. Firstly, our validation of the Kernel-smoothed permutation protocol focuses on its application to 3 predefined closed-form tests. Nonetheless, our methodology has the potential to be extended to test statistics with indeterminate distributions, such as those utilized in the interaction-integrated linear mixed model (Li et al. 2023). Secondly, our current transformation approaches primarily target the third (skewness) and fourth (kurtosis) moments. Future endeavors could involve transformations by higher-order moments and explore their relative efficacy in estimating small *P*-values. Furthermore, we have yet to provide closed-form mathematical derivations that theoretically quantify the superiority of the Kernel-Smoothed Permutation. Finally, the potential of the Kernel-smoothed permutation framework extends beyond our current scope, encompassing a broader range of omics data and testing techniques, making it a versatile tool in genetic studies for approximating small *P*-values. Exploration of these expanded applications remains a subject for forthcoming research.

## Supplementary Material

iyag119_Supplementary_Data

## Data Availability

The gene model file used in SKAT is openly available at https://www.gencodegenes.org/human/release_26.html. The WTCCC dataset is available at https://www.wtccc.org.uk. The SKAT package is available at https://cran.r-project.org/web/packages/SKAT/SKAT.pdf; the Utilities package is available at https://cran.r-project.org/web/packages/utilities/index.html; and the MASS package is available at https://cran.r-project.org/web/packages/MASS/index.html. Bandwidth calculation methods in KDE is available at https://www.rdocumentation.org/packages/stats/versions/3.6.2/topics/bandwidth. Kernel-smoothed permutation is freely available on GitHub under the MIT license and can be found at https://github.com/theLongLab/Kernel_smoothed_permutation. Supplemental material is available at *GENETICS* online.
