# Supplementary material for: Kernel-smoothed permutation for extreme P-value estimation in genetic association studies
Source: Genetics. 2026 May 11;233(3):iyag119. doi: 10.1093/genetics/iyag119 (PMC13334087; doi:10.1093/genetics/iyag119)

**Figure S1.** Comparison between Naïve permutation (red), Kernel-smoothed permutation (green for full samples and blue for 10% subset samples) under the optimal transformation, and the fastPerm method (yellow). *P*-values are compared in absolute -log_10_(*p*-value) difference for a *p*-value accuracy threshold of ${10}^{-7}$ and an optimal bandwidth coefficient of 5. (**A**) represents the two-sample t-test, with the optimal transformation as original (no transformation); (**B**) represents the SKAT (gene *LEMD2*), with the optimal transformation as log transformation; and (**C**) represents the chi-squared test, with the optimal transformation as kurtosis-driven Box-Cox transformation.


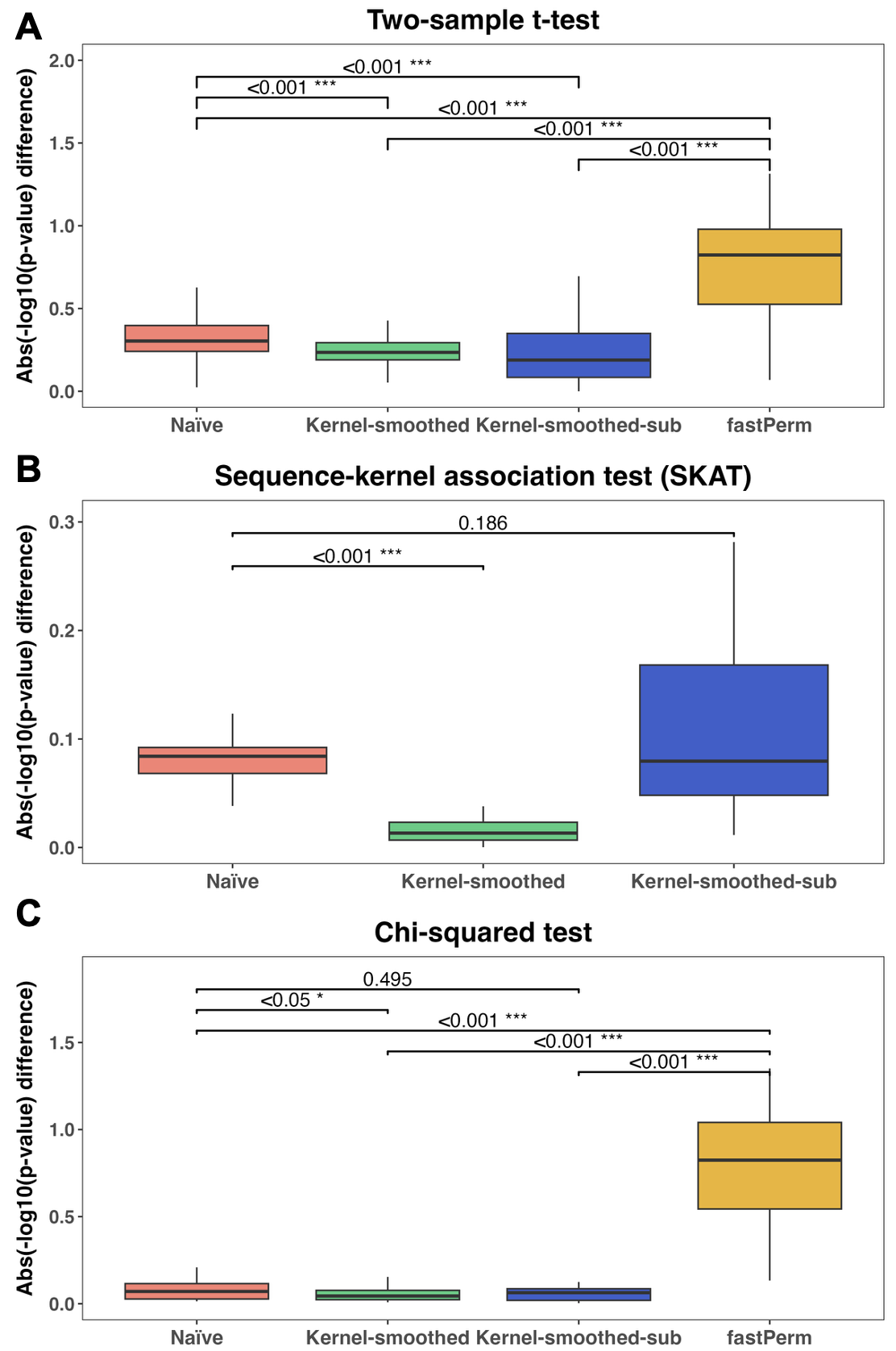


**Figure S2.** Comparison of *p*-values from Kernel-smoothed permutation under different transformations in SKAT (gene *LEMD2*). *P*-values are compared in absolute -log_10_(*p*-value) difference for a *p-*value accuracy threshold of ${10}^{-7}$ and an optimal bandwidth coefficient of 5. (**A**) represents the *p-*value comparison for full samples; (**B**) represents the *p*-value comparison for 10% sub-samples; (**C**) and (**D**) represent the skewness and kurtosis values under different transformations for full samples; and (**E**) and (**F**) represent the density (relocated) plots underlying distributions of various transformations along with their focused visuals of the tails for full samples.


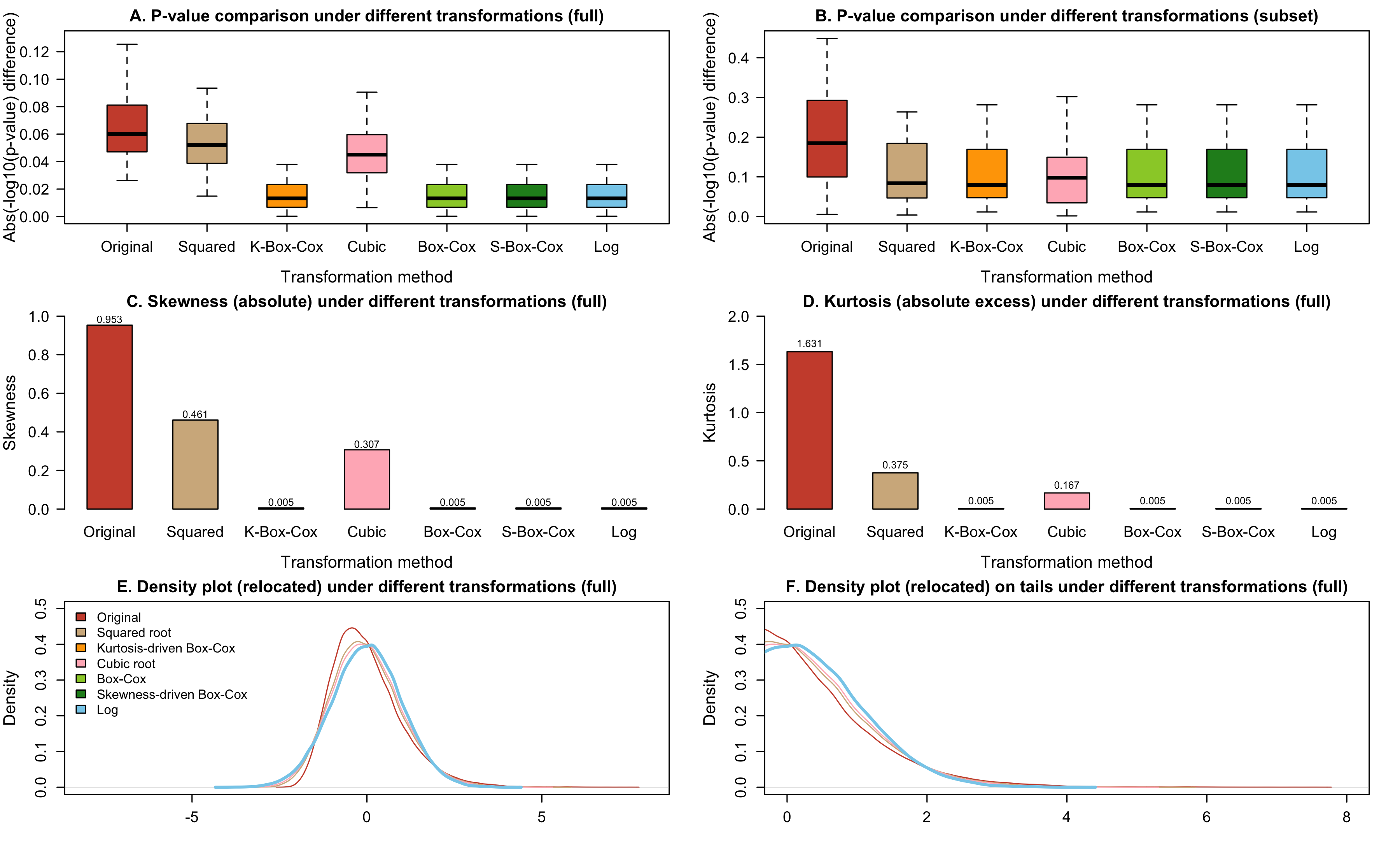


**Figure S3.** Comparison of *p*-values from Kernel-smoothed permutation under different transformations in the chi-squared test. *P*-values are compared in absolute -log_10_(*p*-value) difference for a *p*-value accuracy threshold of ${10}^{-7}$ and an optimal bandwidth coefficient of 5. (**A**) represents the *p*-value comparison for full samples; (**B**) represents the *p*-value comparison for 10% sub-samples; (**C**) and (**D**) represent the skewness and kurtosis values under different transformations for full samples; and (**E**) and (**F**) represent the density (relocated) plots underlying distributions of various transformations along with their focused visuals of the tails for full samples.


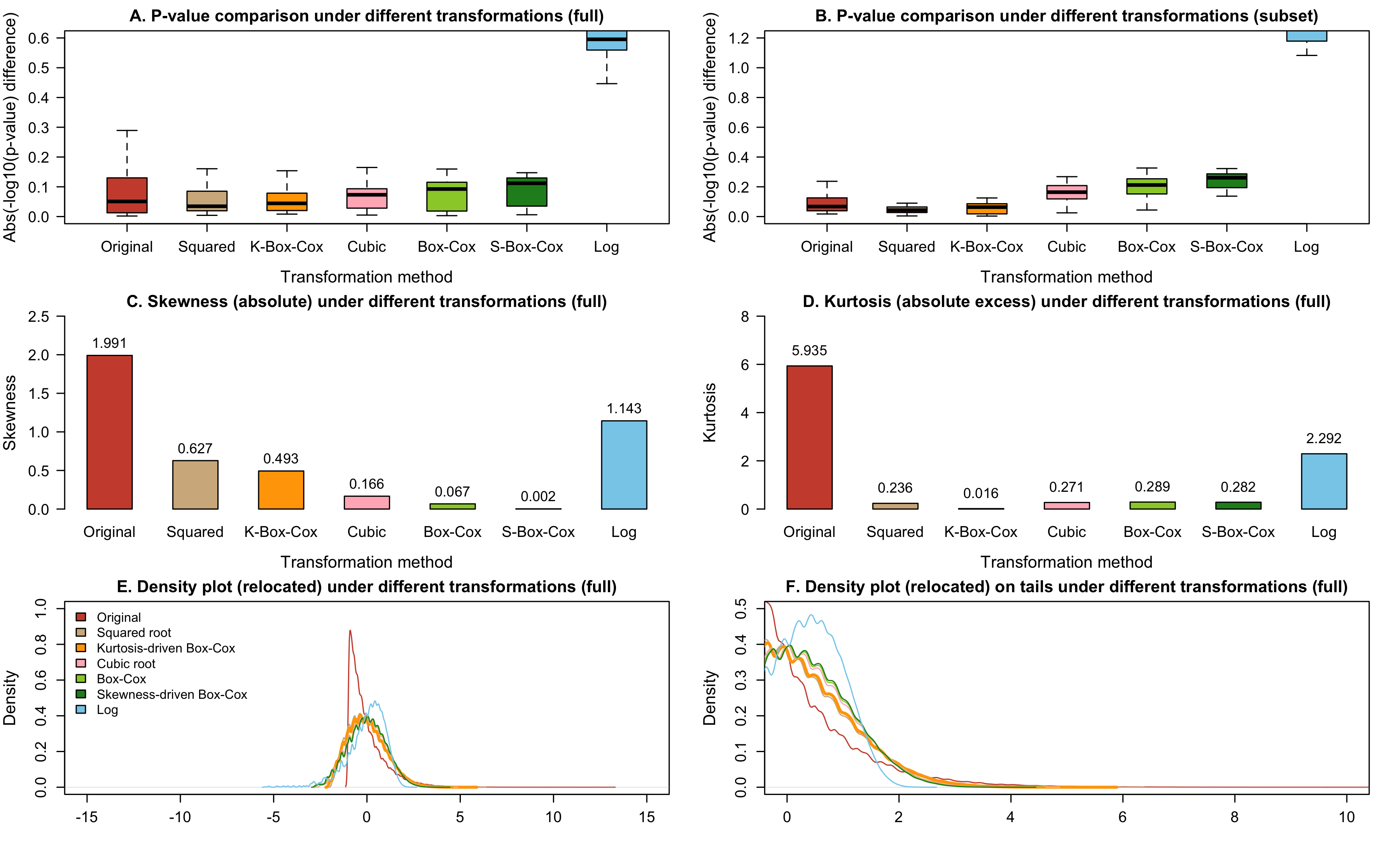


**Figure S4.** Comparison of *p*-values for different bandwidth coefficients by the two-sample t-test at the *p*-value accuracy thresholds of ${10}^{-7}$ and ${10}^{-8}$. *P*-values are compared in absolute -log_10_(*p*-value) difference. (**A-C**) represent the *p*-value comparison for the full samples, 50% sub-samples, and 10% sub-samples at a *p*-value accuracy threshold of ${10}^{-7}$ and the bandwidth coefficient = 3,5,9; (**D-F**) represent the *p*-value comparison for the full samples, 50% sub-samples, and 10% sub-samples at a *p*-value accuracy threshold of ${10}^{-8}$ and the bandwidth coefficient = 3,7,9.


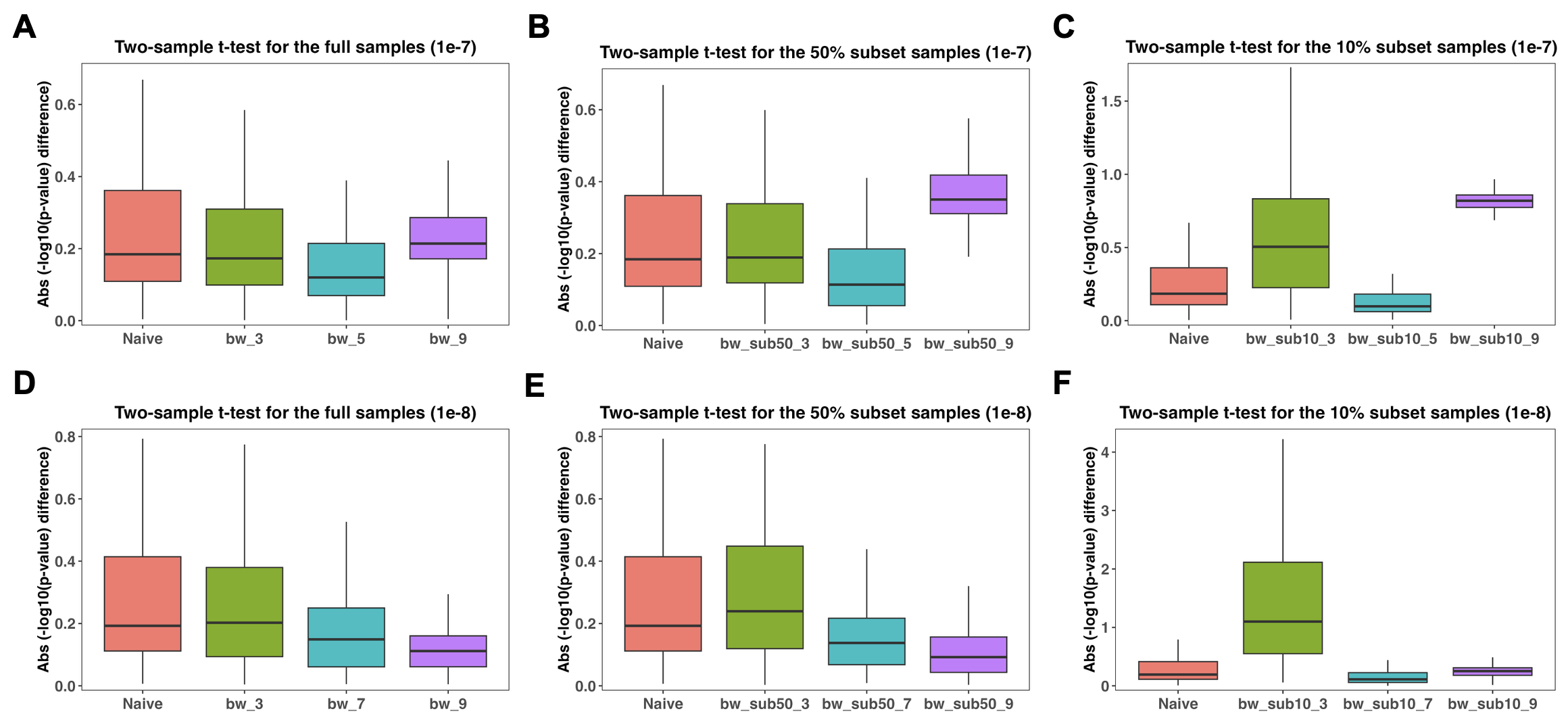


**Figure S5.** Comparison of computational time between Naïve permutation and Kernel-smoothed permutation. (A) represents the computational time of the two permutations for 14 significant SNPs of GWAS-CD with a *p*-value accuracy threshold of ${10}^{-7}$ (B) represents the computational time of the two permutations for 10 significant SNPs of GWAS-CD with a *p*-value accuracy threshold of ${10}^{-8}$.


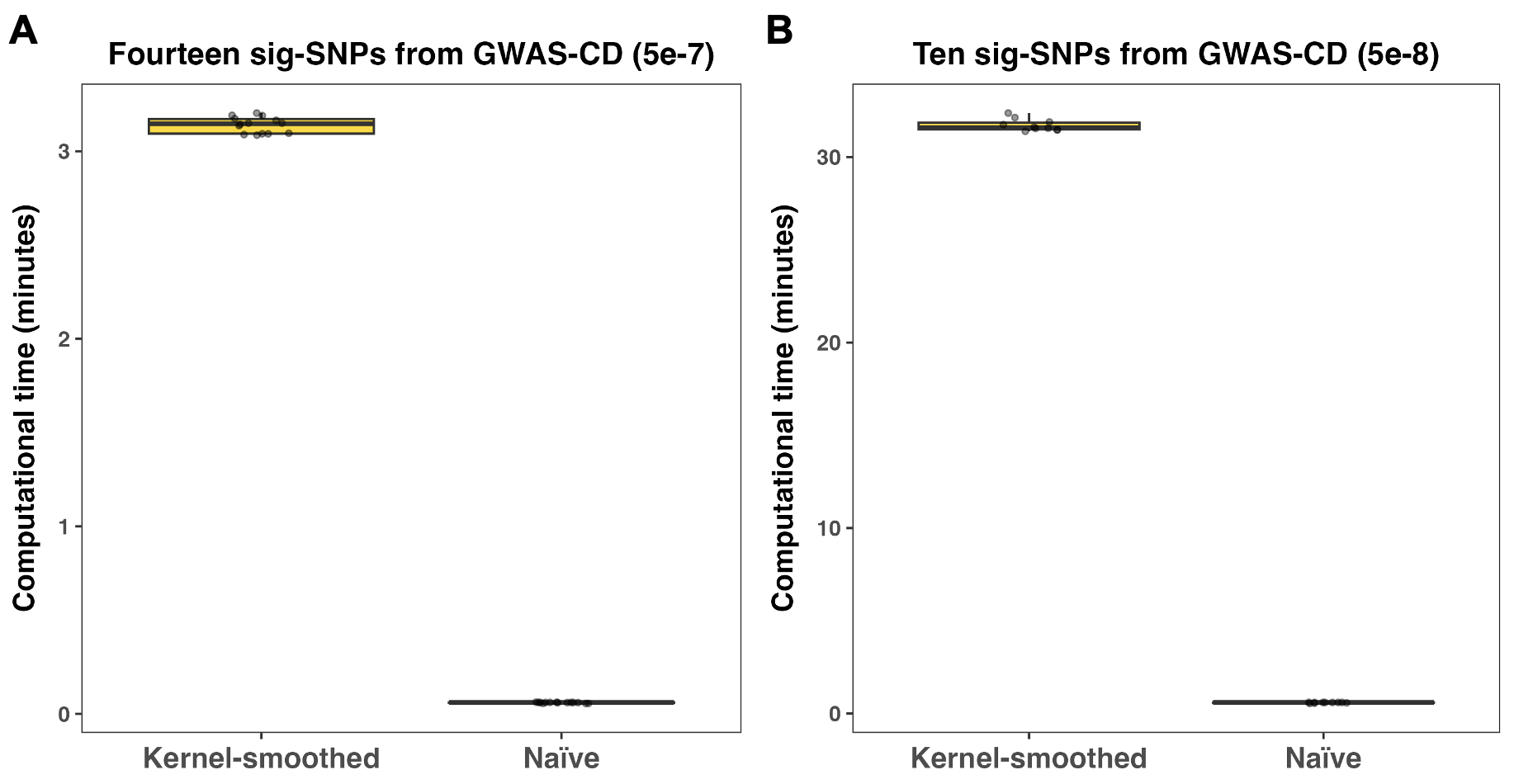

Supplement: iyag119_Supplementary_Data [file iyag119_supplementary_data.zip › Supplementary_Figures_GENETICS-2026-309250.docx]
